# Maintenance of Sperm Variation in a Highly Promiscuous Wild Bird

**DOI:** 10.1371/journal.pone.0028809

**Published:** 2011-12-15

**Authors:** Sara Calhim, Michael C. Double, Nicolas Margraf, Tim R. Birkhead, Andrew Cockburn

**Affiliations:** 1 Section of Ecology, Department of Biology, University of Turku, Turku, Finland; 2 Australian Antarctic Division, Tasmania, Australia; 3 Research School of Biology, Australian National University, Canberra, Australia; 4 Department of Animal and Plant Sciences, University of Sheffield, Sheffield, United Kingdom; University of Western Ontario, Canada

## Abstract

Postcopulatory sexual selection is an important force in the evolution of reproductive traits, including sperm morphology. In birds, sperm morphology is known to be highly heritable and largely condition-independent. Theory predicts, and recent comparative work corroborates, that strong selection in such traits reduces intraspecific phenotypic variation. Here we show that some variation can be maintained despite extreme promiscuity, as a result of opposing, copulation-role-specific selection forces. After controlling for known correlates of siring success in the superb fairy-wren (*Malurus cyaneus*), we found that (a) lifetime extra-pair paternity success was associated with sperm with a shorter flagellum and relatively large head, and (b) males whose sperm had a longer flagellum and a relatively smaller head achieved higher within-pair paternity. In this species extrapair copulations occur in the same morning, but preceding, pair copulations during a female's fertile period, suggesting that shorter and relatively larger-headed sperm are most successful in securing storage (defense), whereas the opposite phenotype might be better at outcompeting stored sperm (offense). Furthermore, since cuckolding ability is a major contributor to differential male reproductive output, stronger selection on defense sperm competition traits might explain the short sperm of malurids relative to other promiscuous passerines.

## Introduction

Female promiscuity is a feature of the breeding system of most passerines, often leading to extrapair paternity (e.g. [1]). Therefore, fitness in males that breed in social pairs but engage in extrapair copulations is the combined success of both extrapair and within-pair siring (e.g. [2]). Sperm competition theory proposes that males are selected to both protect from and overcome a rival male's paternity assurance mechanisms, and that traits involved in paternity defense and offense might be under antagonistic forces [3]–[5]. Several studies have looked at inter-male variation in a given trait and its *concurrent and separate* effect in within-pair and extrapair reproductive success in birds, yet these studies are largely limited to secondary sexual traits (i.e. plumage, song) or age (reviewed in Table S1, n = 20 species). In many cases, the trait has a directional effect on extrapair paternity but no effect on own-nest paternity, with two notable exceptions: crown ultraviolet hue in blue tits (*Cyanistes caeruleus*, [6]) and tail length in cape sugarbirds (*Promerops cafer*, [7]) have opposite effects. Focus on primary sexual traits and male copulation roles has largely been restricted to empirical studies of controlled matings in invertebrates (e.g. [8]–[10]), social status manipulation in domestic fowl (e.g. [11]), hormonal manipulation in a wild passerine [12] and alternative mating tactics in centrarchid fish (e.g. [13], [14]).

Most long-term studies of birds have difficulty measuring lifetime measures of fitness, due to low assignment of extra-pair sires and/or tracking fitness of dispersers (e.g. [1], [15]). Yet, accurate measurement of selection in the wild is best achieved when not restricted to a spatio-temporal snapshot of an otherwise well studied system [16], [17]. Lifetime paternity success provides a direct assessment of differential pre- and postcopulatory success [18]. However, using data from natural, unobserved matings has inherent unknown confounding effects, such as inter-male variation in sperm competition risk (likelihood of being cuckolded), relative timing and number of copulations for a given clutch [19]. The detailed knowledge of the unusual breeding biology of superb fairy-wrens (*Malurus cyaneus*) make this a good species to investigate the evolutionary consequences of sperm morphology variation on extrapair and within-pair siring success in a wild bird. Superb fairy-wrens are territorial, facultative cooperative breeders, with a permanent social pair bond [20], [21]. Males are philopatric and both sexes acquire their social mates passively rather than through selection on phenotype [21]. Males queue for dominance on their natal or adjacent territories [22], while females compete for rare vacancies through dispersal [21]. By contrast, females show strong precopulatory mate choice of extragroup sires, preferring males that acquire the nuptial plumage early, an honest signal of male quality [23]–[26]. Regardless of the quality of their own social partner (e.g. when it molted), all females make pre-dawn extragroup forays to mate with these preferred males, usually two or three days before egg-laying [27], leading to extragroup paternity in almost all broods [28]. Helpers (and/or neighbors) of attractive males gain some of these extragroup fertilizations [29], suggesting a hidden lek effect of dawn chorus displays [30] and/or ‘error-prone’ female choice [29]. Remarkably, unless socially paired to a son, females always copulate with their social partner within thirty minutes of returning from the extragroup foray, and/ or with unrelated helpers (Cockburn & Double, unpublished data). Thus sperm competition is pervasive, a view supported by the relatively large size of the testes, proportion of sperm producing tissue and cloacal protuberance [31]–[33].

Passerine sperm is morphologically complex, with helical shaped heads, a large, single fused mitochondrion wrapped around the flagellum ([34], [35], but see [36] for an exception), and move by rotation along the main axis [37]. Both passerine sperm form and function exhibit considerable additive genetic variance and generally show low condition-dependence ([38]–[40], but see [41] for an exception). Comparative work in birds has shown that, as sperm competition increases, mean sperm length increases, although not linearly [42], [43], and both inter-male and intra-male variation in sperm size is reduced [44]–[46]. Recent studies suggest that while there are significant associations between sperm morphology (absolute flagellum length and relative to head length) and sperm motility across species ([47], but see [48]), these can become uncoupled at the intraspecific level in taxa, especially in species under high sperm competition ([40], [49]–[51] but see [52]). It is therefore reasonable to assume that in taxa under strong sperm competition, one might find certain morphometric traits to be associated with cuckolding success and/or defense. We studied cuckolding success and defense in the superb fairy-wren.

## Methods

This study was conducted on an intensively studied color-banded population at the Australian National Botanical Gardens, Canberra [21], [22], in which the breeding history, age and parentage are known for most birds [21], [23], [27]. Ethical approval for this research was given by the Australian National University Animal Experimentation Ethics Committee (permits: F.BTZ.63.04 and F.BTZ.06.07).

Cuckolding success was measured as the lifetime number of illegitimate offspring that survived to four weeks after fledgling (when census provides the most reliable fitness measure; A. Cockburn, pers. comm.). Cuckolding success was computed as a categorical variable using the total number of extrapair offspring a male sired (see Fig. S1). Since the frequency distribution can be interpreted as bimodal, with modes at zero and 3–4 young (which correspond functionally to siring no young or a single brood of extrapair chicks), and a lowest point at 2 young (Fig. S1). Failing to acquire an extrapair copulation (EPC) or to convert an EPC into an extrapair fertilization, which are impossible to differentiate in our data, reflect low competitiveness. Also, since EPC forays are so time-restricted [27], one can assume that there is little between individual variation in the number or EPCs per bout. Therefore, males producing a single extrapair young can be considered to have low success as a sperm competitor. We therefore managed our data distribution by analyzing cuckolding success as a binomial response, where the two classes were 0–1 and 2+ young. Using this cut-off rather than 0–2 versus 3+ young is further supported, as (i) one of the two males producing two EPO gained 100% success in the brood in which it obtained extrapair paternity and (ii) the other was a subordinate individual that successfully cuckolded within its social group. Therefore, both males can be considered successful sperm competitors. Because male age is a strong predictor of extrapair fertilization success, through its positive effect on the nuptial plumage molt date [23], [26], male breeding experience (i.e. number of breeding seasons it was alive) was included as a covariate in the Generalized Linear Model (glm function with logit link and (quasi)binomial error distribution; n = 59 males). In order to assess the robustness of this analysis, it was repeated using GLM with negative binomial errors (glm.nb function), an alternative interpretation of Fig. S1 distribution (see Supplementary Material for more details). We present the results of the latter in the Supplementary Material). Cuckolding defense success refers to the proportion of sired fledglings in all broods sampled while a given male was dominant. Broods assigned to males while in an incestuous pair with their own mothers were excluded (n = 4 broods), since inbreeding avoidance by females may bias within-nest paternity success [21]. We used Generalized Linear Mixed Models, GLMMs (lmer function with logit link and binomial error distribution), to estimate the fixed effects of sperm morphometric traits, with male identity incorporated as a random factor. Helper number was included as a covariate, since dominant males without helpers are cuckolded less, possibly since paternity assurance increases care [21], [23], [28]. One measure of sperm defense per male (average number of sired young across broods) was not appropriate since the total number of fledglings, female identity and helper number differed across broods. A total of n = 255 broods belonging to 47 males was used.

Sperm samples were collected non-invasively by collecting the liquid part of the faeces (see [53]) from n = 59 adult males in December 2005. This method [53] has been shown to provide reliable sperm morphometry data. Sperm morphometry was measured using digital imaging software (Leica IM50) and photographs taken using light microscopy. Three independent sperm traits were directly measured (flagellum, head and straight midpiece lengths) and three composite traits were calculated (total length, flagellum∶head and midpiece∶flagellum length ratios) to the nearest 0.1 µm (for more details see [38] and [47]). In order to minimize autocorrelation between predictor variables, independent and composite sperm traits were tested separately (note that independent traits were not correlated with each other, r<|0.17| and p>0.2). Although five sperm per male are generally used to describe sperm morphometry in passerine birds ([38]), we included all sampled males in the analysis irrespective of number of sperm measured each (mean = 7 sperm per male, range = 1 to 10; see Fig. S2) since this species has low intra-male variation (Table S2), reasonable intra-male repeatability ([45]; Table S2), and a single sperm captures c. 70% of this intra-male variation (see [54]; Fig. S3). In fact, the superb fairy-wren shows a two fold difference between inter- and intra-male coefficient of variation for sperm length, one of the highest for which comparable variation indices are available (inter∶intra CV ratio = 1.9; range in 26 species = 0.8 to 2.1; [44]–[46]). Nonetheless, we conservatively weighted all analyses by sampling effort category (low weight given to males with fewer than five sperm measured). Although we cannot report across-year repeatability in sperm traits, it is unlikely that sperm sampling restriction to 2005 would significantly confound our results for the following three reasons. First, in contrast to the only study that has shown environmentally-induced plasticity in sperm morphology in a passerine [41], there is no evidence that primary sexual traits and hormonal profiles at the time of sperm production differ across males of different social status or social group in this species (e.g. [25], [31]). Second, we found no variation in any sperm morphometric trait with respect to age, status or group composition at the time of sampling (MANOVA, p>0.2, Table S3). Third, evidence from a two passerine species suggests considerable within-individual across-year repeatability (*Agelaius phoeniceus*, S Lüpold, pers. comm.; *Troglodytes aedon*, E. Cramer, pers. comm.). Nonetheless, all analyses were repeated with the subsample of males with at least 5 sperm measured (see Table S4).

Finally, the inclusion of males that are potentially still reproductively active past the 2008–9 breeding season, the last year we have completed paternity assignment data (n = 20/59 and n = 17/47 males for extrapair and within-pair success, respectively) create a further and potentially confounding factor in our sample. We highlight the possibly biased data points in the Figures and provide the results using a restricted dataset excluding those males in Table S4. Model simplification was achieved using stepwise removal of terms and comparison of alternative models' fit using likelihood ratio tests [55]. Quasibinomial error structure was used in cases where overdispersion needed to be accounted for [55]. All analyses were conducted in R version 2.10.1 (R Development Core Team). Effect sizes and their 95% confidence intervals [56] were calculated for each alternative data subset's minimal adequate final models, based on the standardized variable methods proposed by [57] (see Table S5).

## Results

**Figure 1:**
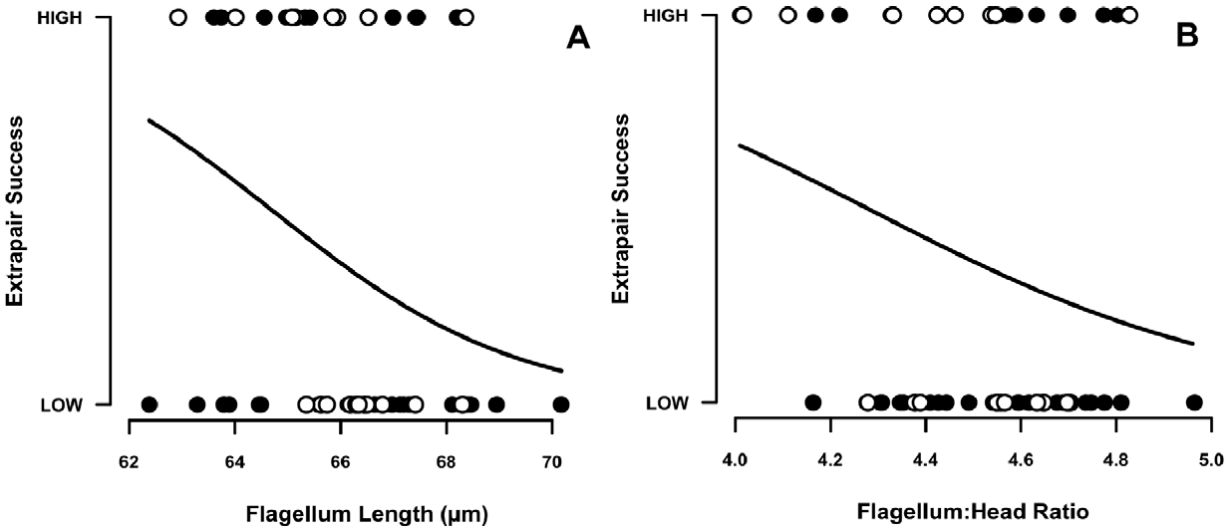
Negative associations between extrapair reproductive success and sperm morphology. (**A**) flagellum length and (**B**) relative length of flagellum to head section. Extrapair paternity success was transformed into a categorical variable based on the observed binomial frequency distribution peaks of fledged extrapair sired young (low = one or fewer, high = two or more). Males that are potential active breeders past the date of the current paternity assessment (2008/9 season) are represented by the open circles (n = 20 of the total n = 59 males). Fitted curves were calculated using the regression estimates from fitted models (male breeding experience included as a covariate).

After controlling for the positive effect of male breeding experience (i.e. breeding season number), success at siring extrapair offspring declined with both flagellum length (Fig. 1; GLM with quasibinomial errors; season number: estimate (± s.e.) = 0.98±0.27, z = 3.64, p = 0.0006; flagellum length: estimate (± s.e.) = −0.67–44.83±16.56, z = −2.71, p = 0.009; n = 59 males) and flagellum∶head length ratio (Fig. 1; GLM with quasibinomial errors; season number: estimate (± s.e.) = 0.90±0.26, z = 3.43, p = 0.001; flagellum∶head ratio: estimate (± s.e.) = −18.59±9.23, z = −2.01, p = 0.049, n = 59 males). Therefore, cuckolding success was associated with sperm with a shorter flagellum and relatively larger heads. The alternative negative binomial GLM method shows these results are robust (Table S4). Removal of males that could be reproductively active past the end date for paternity data (n = 12 males who could change from low to high extrapair success late in their lives), did not qualitatively change the results (flagellum length p = 0.018; flagellum∶head ratio p = 0.048; Table S4 and Table S5). The same was true for the restriction of the dataset to males with at least five measured (Table S4 and Table S5).

**Figure 2:**
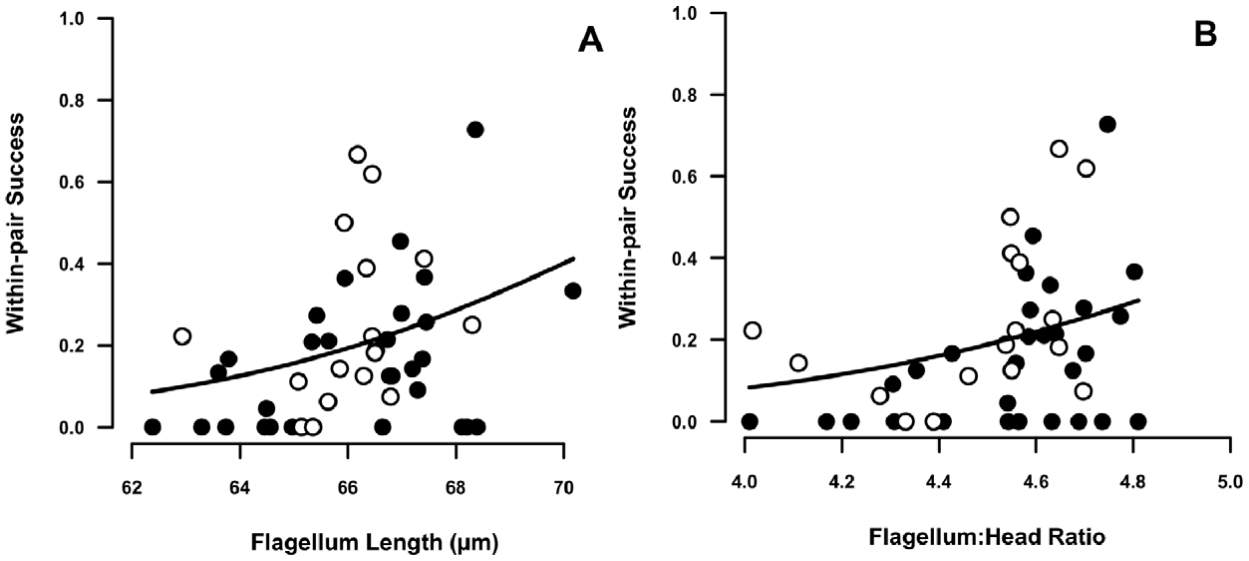
Positive associations between within-pair reproductive success and sperm morphology. (**A**) flagellum length and (**B**) relative length of flagellum to head section. Within-pair reproductive success is measured as the proportion of young at a social dominant male's nest(s). Analyses were conducted using male identity as a random factor (i.e. mixed models). Plots refer to mean paternity for all broods sampled. Males that are potential active breeders past the date of the current paternity assessment (2008/9 season) are represented by the open circles (n = 17 of the total n = 47 males). Fitted curves were calculated using the regression estimates from non-mixed effects fitted models.

In contrast, males with sperm with a longer flagellum and relatively shorter heads were more successful at preventing cuckoldry (Fig. 2; binomial GLMMs with male identity as a random factor; flagellum length: estimate (± s.e.) = 20.08±8.31, z = 2.42, p = 0.016; flagellum∶head ratio: estimate (± s.e.) = 11.50±4.31, z = 2.67, p = 0.008; n = 255 broods assigned to 47 males). The number of helpers did not affect within-brood paternity success in this sample (p>0.2). Although our measure of cuckolding avoidance (proportion of fledgling sired in all assigned nests) can be biased either way by future potential breeding attempts (n = 17 dominant males alive past the last available paternity analysis), removal of these data points did not change any of the previous results (flagellum length p = 0.03; flagellum∶head ratio p = 0.008). Note, however, that restriction of the dataset to males with at least five measured sperm considerably decreased effect size estimates (c. by one third) and rendered the results not significant for either sperm trait (Table S4 and Table S5).

Midpiece size (absolute or relative to flagellum length) was not associated with either extra- or within-pair reproductive success (all models, p>0.4; see Table S4). In summary, the same sperm morphometric traits have opposite effects for cuckoldry success and cuckoldry avoidance.

## Discussion

We found evidence of opposite selection on male sperm traits in superb fairy-wrens, a species under intense sperm competition. Male fairy wrens with shorter flagella and relatively larger heads sired more extrapair offspring, but were less likely to secure paternity at their own nest than males with the opposite sperm phenotype. To our knowledge, this is the first evidence for a naturally occurring selective trade-off for sperm morphology in a wild population.

Sperm size, design and numbers are known to influence the outcome of sperm competition (e.g. reviewed in [18], [58], [59]). Fertilization efficiency of sperm is a complex trait that is further influenced by female (cryptic) choice processes, often mediated by ejaculate storage [60], [61]. Although we currently lack data on female fairy-wren sperm storage morphology, we can speculate on possible mechanisms that explain the association between sperm design and reproductive success based on what we know about (i) the likely sperm competition processes operating in this species, (ii) the differences in the timing and number of copulations between the pair and the extrapair mate, and (iii) the unique features of passerine sperm morphology.

### Sperm competition context

The mating system of fairy-wrens theoretically generates a sperm competition scenario that is analogous to a random role (i.e. no strict assignment of males to the offense or defense position in the interaction), possibly loaded raffle (i.e. one male's sperm is devalued), with negligible sperm limitation, and very high risk of sperm competition for both the extrapair and the pair male (e.g. [62]). Females seek extrapair copulations regardless of the quality of their own mate [27], and unless paired to their son, always copulate with the social pair male as well (Cockburn & Double, unpublished data). Moreover, the high rate of sperm production [63], [64], large testes and cloacal protuberance [31]–[33], relatively low breeding synchrony and hence intensity of extrapair matings [25], [30], and similar levels of circulating testosterone and sperm reserves between dominant males during the breeding season [25], [31], suggest that sperm limitation may be negligible. In this scenario, males are theoretically predicted to invest equally in sperm numbers irrespective of their perceived role (i.e. offense or defense) at the time of copulation [65]–[67]. Therefore, we can assume that adaptive ejaculate allocation (e.g. reviewed in [68]) is unlikely to influence sperm competition outcome in this species. Moreover, the strong directional selection on a honest phenotypic signal (nuptial plumage moult date, e.g. [26]) suggests that sperm competition processes based on genetic (in)compatibility ‘loaded raffle’ (e.g. [69]) are also an unlikely confound in this system. In addition, the timing of extrapair copulations is fairly consistent across females (see below).

### Timing of mating

Rival males copulate at the same time relative to ovulation. Extragroup copulations are always sought by females three days prior to egg laying [27]. Pair males follow their females closely on her return from the foray, and they copulate with her within half an hour of her return (Cockburn & Double, unpublished data). Thereafter, males show little interest in mate-guarding and/or copulation, and instead spend large part of the day displaying to females on neighbouring territories [70]. Potential ‘loading’ in such sperm competition raffle is thus intimately associated with the relative timing of copulation, with the extragroup male always first to inseminate the female. Paradoxically, when discussing sperm-female interactions mechanisms at the proximate level (see below), the extrapair and pair males exert the roles of paternity defender and offender, respectively: the within-pair male tries to overcome the extrapair male's previously stored ejaculate. At the ultimate level, securing within-pair paternity in species with paternal care is inherently a defensive fitness strategy.

### Sperm morphology and female-sperm interactions

Sperm design, i.e. the relative lengths of sperm components rather than total length or absolute component size, might be important target of selection, especially when they provide better correlates of sperm function [71]. For instance, the relative size of the head to the flagellum (i.e. drag vs. power) was proposed [71] and later found ([40], [47], [51], but see [48]) to be a good predictor of sperm velocity in birds: sperm with higher flagellum∶head ratios swam faster, although this relationship did not hold at the intraspecific level in three promiscuous species (*Agelaius phoeniceus*, [49]; *Quelea quelea*, [50]; *Tachycineta bicolor*, [52]). Nevertheless, the pre- and post-copulatory scenarios where selection acts in our study species are quite different (e.g. EPC-seeking by all females and lack of precopulatory pair choice; see Introduction) from the more ‘traditional’ mating ecology of the three other promiscuous species aforementioned. More data are therefore needed. The relationship between flagellum and midpiece lengths has also been considered an important trait, associated with sperm energetic dynamics and sperm function [71]–[73]. It is interesting to note that in the present study neither absolute midpiece length nor its ratio to flagellum length were found to be associated with either type of paternity success. However, (relative) midpiece size is not as closely linked to sperm function in passerine birds ([40], [48], [51], cf. [47]) as in chickens or mammals (e.g. [74], [75]), which might explain its lack of relationship with fitness.

Passerine sperm are stored in sperm storage tubules (SSTs), located at the utero-vaginal junction of the female reproductive tract, with their heads facing the distal blind-ended part [76], [77]. Across bird species, sperm total length generally increases with sperm competition level, but the latter relationship is the indirect result of a stronger sperm-length-SST-length correlation [42]. In fact, the role of sperm-female interactions in the evolution of male gametes is clear and well supported empirically (reviewed in [61]). The most detailed histological study of SSTs in another highly promiscuous passerine, the alpine accentor, *Prunella collaris* [78], found no evidence for contractile elements, which would provide direct female anatomical control mechanisms over sperm access to and persistence in the SSTs. Therefore, although we can not rule out possible biochemical processes, it is not unrealistic to assume that features of passerine sperm themselves might strongly influence access to and endurance within the SSTs. For instance, relatively longer heads (e.g. more twists in the helix) might improve a sperm's resistance to passive loss from the SSTs, thus benefiting the extrapair male. Sperm with a longer flagellum and relatively shorter heads are predicted to have higher thrust forces and reduced drag [71], [79], often explaining the associated *higher in vitro* velocity [47]. However, it is unclear how higher thrust benefits sperm in the offense capacity, since (i) sperm velocity and flagellum∶head ratio are not correlated across males of promiscuous species (see Introduction), (ii) there is no evidence for active sperm displacement in birds [19], [80], and (iii) it has been shown that avian sperm are passively transported from the SSTs to the site of fertilization, the infundibulum [81]. We can speculate that more powerful and/or energy efficient (if not faster) morphometry might increase the proportion of within-pair male sperm that enter the SSTs, or perhaps those SSTs placed higher in the reproductive tract, and thus closer to the site of fertilization, or reduce the rate at which sperm are lost from the SSTs, as was observed in the domestic fowl (*Gallus g. domesticus*, [82]).

### The short sperm of Maluridae

The evolution of sperm morphology in a within-species context has been the focus of several empirical (e.g. reviewed in [58]) and theoretical studies (e.g. [65], [66], [83]). For instance, longer sperm increase the competitive potential of an ejaculate or promote female sperm choice because longer sperm may, among other reasons, swim faster, live longer, be more effective in sperm displacement within the female reproductive tract, or indicate higher male quality [58]. On the other hand, shorter sperm might be favored under raffle processes if the same number of sperm can be invested into an ejaculate for reduced energetic (and/or spatial) cost (e.g. [66]). Recent theoretical and comparative work suggest that the typical mode of sperm competition in birds follows raffle principles [43], [83], and the latter also applies to fairy-wrens (see above). Cuckolding success (cf. avoidance) is likely to be the major cause of male differential reproductive success in this species (cf. other passerines; e.g. [84]) since: (i) it is mostly under female control [27], (ii) a very small percentage (4–5%) of males sire the majority of extrapair offspring in the population (33–47% [28], this study), (iii) most dominant males suffer some within-pair paternity loss (77–95% broods have at least one extrapair chick [28], this study) and one third of males sire none of the young raised on their territory (13/46, 38%; this sample), (iv) subordinate male direct fitness is mostly derived through extragroup cuckoldry, particularly as they never mate with their mother [21], [30], [85]. Since shorter sperm was positively associated with greater cuckolding success, this might explain why Maluridae has relatively shorter sperm than expected for their body and relative testes sizes [43]. Moreover, producing shorter sperm might be a consequence of the selection for higher proportion of sperm producing tissue [33] in already space-constrained testes.

### Conclusion

The observed antagonistic selection forces acting on superb fairy-wren sperm morphology provide a feasible mechanism for maintenance of some morphological variation under extreme postcopulatory sexual selection and preclude the existence of a universally favorable sperm phenotype at any given breeding season (cf. sexually-selected sperm hypothesis; [86]). Moreover, this study attests to the value of using well-documented, long-term study systems to improve our understanding of sperm competition evolutionary processes in natural conditions. We recommend that future work on this, and other (promiscuous) wild taxa should focus on proximate level enquiries, including female reproductive morphology and the genetic basis and covariation between sperm form and function.

## Supporting Information

Figure S1 **Frequency distribution of number of extrapair offspring per male.**
(PDF)

Figure S2 **Frequency distribution of the number of sperm measured per male.**
(PDF)

Figure S3 **Assessment of the accuracy of individual sperm morphometry estimates obtained using different numbers of sperm per male.**
(PDF)

Table S1 **Review of the evidence for paternity success trade-offs in male (pre-copulatory) phenotype in birds.**
(PDF)

Table S2 **Repeatability of sperm measurements.**
(PDF)

Table S3 **Effects of status, age and group type in sperm morphometrics.**
(PDF)

Table S4 **Full statistical outputs.**
(PDF)

Table S5 **Effect sizes.**
(PDF)

## References

[1] Griffith SC, Owens IPF, Thuman KA (2002). Extra pair paternity in birds: a review of interspecific variation and adaptive function.. Mol Ecol.

[2] Webster M, Pruett-Jones S, Westneat D, Arnold S (1995). Measuring the effects of pairing success, extra-pair copulations and mate quality on the opportunity for sexual selection.. Evolution.

[3] Parker G (1970). Sperm competition and its evolutionary consequences in the insects.. Biol Rev.

[4] Parker G (1970). The reproductive behavior and the nature of sexual selection in Scatophaga stercoraria L. (Diptera: Scatophagidae). VII. The origin and evolution of the passive phase.. Evolution.

[5] Parker G, Smith R (1984). Sperm competition and the evolution of animal mating strategies.. Sperm Competition and the Evolution of Animal Mating Strategies.

[6] Delhey K, Johnsen A, Peters A, Andersson S, Kempenaers B (2003). Paternity analysis reveals opposing selection pressures on crown coloration in the blue tit (Parus caeruleus).. Proc R Soc Lond B.

[7] Mcfarlane ML, Evans MR, Feldheim KA, Preault M, Bowie RC (2010). Long tails matter in sugarbirds–positively for extrapair but negatively for within-pair fertilization success.. Behav Ecol.

[8] Civetta A, Clark AG (2000). Correlated effects of sperm competition and postmating female mortality.. Proc Nat Acad Sci.

[9] Nilsson T, Fricke C, Arnqvist G (2003). The effects of male and female genotype on variance in male fertilization success in the red flour beetle (Tribolium castaneum).. Behav Ecol Sociobiol.

[10] House CM, Simmons LW (2006). Offensive and defensive sperm competition roles in the dung beetle Onthophagus taurus (Coleoptera : Scarabaeidae).. Behav Ecol Sociobiol.

[11] Cornwallis CK, Birkhead T (2006). Social status and availability of females determine patterns of sperm allocation in the fowl.. Evolution.

[12] Mcglothlin JW, Whittaker DJ, Schrock SE, Gerlach NM, Jawor JM (2010). Natural selection on testosterone production in a wild songbird population.. Am Nat.

[13] Neff BD (2003). Sperm investment and alternative mating tactics in bluegill sunfish (Lepomis macrochirus).. Behav Ecol.

[14] Burness G, Casselman SJ, Schulte-Hostedde AI, Moyes C, Montgomerie R (2004). Sperm swimming speed and energetics vary with sperm competition risk in bluegill ( Lepomis macrochirus ).. Behav Ecol Sociobiol.

[15] Griffith S, Jamieson BG (2007). Intra and extrapair paternity.. Reproductive biology and phylogeny of birds.

[16] Cornwallis C, Uller T (2010). Towards an evolutionary ecology of sexual traits.. Trends Ecol Evol.

[17] Sardell RJ, Arcese P, Keller LF, Reid JM (2011). Sex-specific differential survival of extra-pair and within-pair offspring in song sparrows, Melospiza melodia.. P Roy Soc B-Biol Sci.

[18] Pizzari T, Birkhead T (2002). The sexually-selected sperm hypothesis: sex-biased inheritance and sexual antagonism.. Biol Rev.

[19] Birkhead T (1998). Sperm competition in birds.. Rev Reprod.

[20] Double MC, Peakall R, Beck NR, Cockburn A (2005). Dispersal, philopatry, and infidelity: Dissecting local genetic structure in superb fairy-wrens (Malurus cyaneus).. Evolution.

[21] Cockburn A, Osmond H, Mulder RA, Green DJ, Double MC (2003). Divorce, dispersal and incest avoidance in the cooperatively breeding superb fairy-wren Malurus cyaneus.. J Anim Ecol.

[22] Cockburn A, Osmond H, Mulder R, Double MC, Green DJ (2008). Demography of male reproductive queues in cooperatively breeding superb fairy-wrens Malurus cyaneus.. J Anim Ecol.

[23] Dunn PO, Cockburn A (1999). Extrapair mate choice and honest signaling in cooperatively breeding superb fairy-wrens.. Evolution.

[24] Peters A, Astheimer L, Boland C, Cockburn A (2000). Testosterone is involved in acquisition and maintenance of sexually selected male plumage in superb fairy-wrens, Malurus cyaneus.. Behav Ecol Sociobiol.

[25] Peters A, Astheimer L, Cockburn A (2001). The annual testosterone profile in cooperatively breeding superb fairy-wrens, Malurus cyaneus, reflects their extreme infidelity.. Behav Ecol Sociobiol.

[26] Cockburn A, Osmond H, Double MC (2008). Swingin' in the rain: condition dependence and sexual selection in a capricious world.. Proc R Soc Lond B.

[27] Double M, Cockburn A (2000). Pre-dawn infidelity: females control extra-pair mating in superb fairy-wrens.. Proc R Soc Lond B.

[28] Mulder R, Dunn P, Cockburn A, Lazenbycohen K, Howell M (1994). Helpers liberate female fairy-wrens from constrains on extra-pair mate choice.. P Roy Soc Lond B Bio.

[29] Double M, Cockburn A (2003). Subordinate superb fairy-wrens (Malurus cyaneus) parasitize the reproductive success of attractive dominant males.. P Roy Soc Lond B Bio.

[30] Cockburn A, Dalziell AH, Blackmore CJ, Double MC, Kokko H (2009). Superb fairy-wren males aggregate into hidden leks to solicit extragroup fertilizations before dawn.. Behav Ecol.

[31] Mulder R, Cockburn A (1993). Sperm competition and the reproductive anatomy of male superb fairy-wrens.. Auk.

[32] Rowe M, Pruett-Jones S (2006). Reproductive biology and sperm competition in Australian fairy-wrens.. Avian Poult Biol Rev.

[33] Rowe M, Pruett-Jones S (2011). Sperm Competition Selects for Sperm Quantity and Quality in the Australian Maluridae.. PLoS ONE.

[34] McFarlane RW (1963). The taxonomic significance of avian sperm.. Proc XIII Internat Ornithol Congress.

[35] Pitnick S, Hosken D, Birkhead T, Birkhead T, Hosken D, Pitnick S (2009). Sperm morphological diversity.. Sperm biology.

[36] Birkhead TR, Immler S, Pellatt EJ, Freckleton R (2006). Unusual sperm morphology in the Eurasian Bullfinch (Pyrrhula pyrrhula).. Auk.

[37] Vernon GG, Woolley DM (1999). Three-dimensional motion of avian spermatozoa.. Cell Mot Cytoskel.

[38] Birkhead TR, Pellatt EJ, Brekke P, Yeates R, Castillo-Juarez H (2005). Genetic effects on sperm design in the zebra finch.. Nature.

[39] Simmons LW, Moore AJ, Birkhead TR, Hosken DJ, Pitnick S (2009). Evolutionary quantitative genetics of sperm.. Sperm Biology: An Evolutionary Perspective.

[40] Mossman J, Slate J, Humphries S, Birkhead T (2009). Sperm Morphology and Velocity Are Genetically Codetermined in the Zebra Finch.. Evolution.

[41] Immler S, Pryke S, Birkhead T, Griffith S (2010). Pronounced within-individual plasticity in sperm morphometry across social environments.. Evolution.

[42] Briskie JV, Montgomerie R, Birkhead TR (1997). The evolution of sperm size in birds.. Evolution.

[43] Immler S, Pitnick S, Parker GA, Durrant KL, Lüpold S (2011). Resolving variation in the reproductive tradeoff between sperm size and number.. Proc National Acad Sci.

[44] Calhim S, Immler S, Birkhead TR, Pizzari T (2007). Postcopulatory Sexual Selection Is Associated with Reduced Variation in Sperm Morphology.. PLoS ONE.

[45] Immler S, Calhim S, Birkhead TR (2008). Increased postcopulatory sexual selection reduces the intramale variation in sperm design.. Evolution.

[46] Kleven O, Laskemoen T, Fossøy F, Robertson RJ, Lifjeld JT (2008). Intraspecific variation in sperm length is negatively related to sperm competition in passerine birds.. Evolution.

[47] Lüpold S, Calhim S, Immler S, Birkhead TR (2009). Sperm morphology and sperm velocity in passerine birds.. Proc Biol Sci.

[48] Kleven O, Fossøy F, Laskemoen T, Robertson RJ, Rudolfsen G (2009). Comparative evidence for the evolution of sperm swimming speed by sperm competition and female sperm storage duration in passerine birds.. Evolution.

[49] Lüpold S, Linz GM, Birkhead TR (2009). Sperm design and variation in the New World blackbirds (Icteridae).. Behav Ecol Sociobiol.

[50] Calhim S (2008). Evolutionary significance of intraspecific variation in male passerine reproductive traits (PhD Thesis)..

[51] Helfenstein F, Podevin M, Richner H (2010). Sperm morphology, swimming velocity, and longevity in the house sparrow Passer domesticus.. Behav Ecol Sociobiol.

[52] Laskemoen T, Kleven O, Fossoy F, Robertson RJ, Rudolfsen G (2010). Sperm quantity and quality effects on fertilization success in a highly promiscuous passerine, the tree swallow Tachycineta bicolor.. Behav Ecol Sociobiol.

[53] Immler S, Birkhead T (2005). A non-invasive method for obtaining spermatozoa from birds.. Ibis.

[54] Pattarini JA, Starmer WT, Bjork A, Pitnick S (2006). Mechanisms underlying the sperm quality advantage in Drosophila melanogaster.. Evolution.

[55] Crawley MJ (2007). The R Book.

[56] Nakagawa S, Cuthill IC (2007). Effect size, confidence interval and statistical significance: a practical guide for biologists.. Biol Rev.

[57] Schielzeth H (2010). Simple means to improve the interpretability of regression coefficients.. Methods Ecol Evol.

[58] Snook R (2005). Sperm in competition: not playing by the numbers.. Trends Ecol Evol.

[59] Pizzari T, Parker GA, Birkhead TR, Hosken DJ, Pitnick S (2009). Sperm competition and sperm phenotype.. Sperm Biology: An Evolutionary Perspective.

[60] Eberhard WG (1996). Female Control: Sexual Selection by Cryptic Female Choice.

[61] Pitnick S, Wolfner MF, Suarez SS, Birkhead TR, Hosken DJ, Pitnick S (2009). Ejaculate-female and sperm-female interactions.. Sperm Biology: An Evolutionary Perspective.

[62] Parker G (1990). Sperm competition games - raffles and roles.. P Roy Soc Lond B Bio.

[63] Tuttle E, Pruett-Jones S, Webster M (1996). Cloacal protuberances and extreme sperm production in Australian fairy-wrens.. P Roy Soc Lond B Bio.

[64] Tuttle E, Pruett-Jones S (2004). Estimates of extreme sperm production: morphological and experimental evidence from reproductively promiscuous fairy-wrens (Malurus).. Anim Behav.

[65] Parker G (1990). Sperm competition games: sneaks and extra-pair copulations.. P Roy Soc Lond B Bio.

[66] Parker G (1993). Sperm competition games: sperm size and number under adult control.. P Roy Soc Lond B Bio.

[67] Ball MA, Parker GA (2000). Sperm competition games: A comparison of loaded raffle models and their biological implications.. J Theor Biol.

[68] Wedell N, Gage M, Parker G (2002). Sperm competition, male prudence and sperm-limited females.. Trends Ecol Evol.

[69] Griffith SC, Immler S (2009). Female infidelity and genetic compatibility in birds: the role of the genetically loaded raffle in understanding the function of extrapair paternity.. J Avian Biol.

[70] Green DJ, Cockburn A, Hall ML, Osmond H, Dunn PO (1995). Increased opportunities for cuckoldry may be why dominant male fairy-wrens tolerate helpers.. Proc R Soc Lond B.

[71] Humphries S, Evans JP, Simmons LW (2008). Sperm competition: linking form to function.. Bmc Evol Biol.

[72] Cardullo RA, Baltz JM (1991). Metabolic regulation in mammalian sperm: mitochondrial volume determines sperm length and flagellar beat frequency.. Cell Motil Cytoskel.

[73] Cummins JM, Birkhead TR, Hosken DJ, Pitnick S (2009). Sperm motility and energetics.. Sperm Biology: An Evolutionary Perspective.

[74] Froman DP, Kirby JD (2005). Sperm mobility: Phenotype in roosters (Gallus domesticus) determined by mitochondrial function.. Biol Reprod.

[75] Malo AF, Gomendio M, Garde J, Lang-Lenton B, Soler AJ (2006). Sperm design and sperm function.. Biol Lett-Uk.

[76] Birkhead TR (1987). Sperm-storage glands in a passerine: the zebra finch Poephila guttata (Estrildidae).. J Zool Lond.

[77] Briskie JV, Montgomerie R (1993). Patterns of sperm storage in relation to sperm competition in passerine birds.. Condor.

[78] Chiba A, Nakamura M (2001). Microscopic structure of the sperm storage tubules in the polygynandrous alpine accentor, Prunella collaris (Aves).. Acta Zool-Stockholm.

[79] Katz DF, Drobnis EZ, Bavister BD, Cummins J, Roldan ERS (1990). Analysis and interpretation of the forces generated by spermatozoa.. Fertilization in mammals.

[80] Birkhead T, Biggins J (1998). Sperm competition mechanisms in birds: models and data.. Behav Ecol.

[81] Brillard J (1993). Sperm storage and transport following natural mating and artificial insemination.. Poult Sci.

[82] Froman DP, Pizzari T, Feltmann AJ, Castillo-Juarez H, Birkhead TR (2002). Sperm mobility: mechanisms of fertilizing efficiency, genetic variation and phenotypic relationship with male status in the domestic fowl, Gallus gallus domesticus.. Proc R Soc Lond B.

[83] Parker G, Immler S, Pitnick S, Birkhead T (2010). Sperm competition games: sperm size (mass) and number under raffle and displacement, and the evolution of P2.. J Theor Biol.

[84] Whittingham LA, Dunn PO (2005). Effects of extra-pair and within-pair reproductive success and the opportunity for selection in birds.. Behav Ecol.

[85] Double MC, Cockburn A (2003). Subordinate superb fairy-wrens (Malurus cyaneus) parasitize the reproductive success of attractive dominant males.. Proc R Soc Lond B.

[86] Keller L, Reeve HK (1995). Why do females mate with multiple males? The sexually-selected sperm hypothesis.. Adv Stud Behav.

