# Supplementary material for: Maintenance of Sperm Variation in a Highly Promiscuous Wild Bird
Source: PLoS One. 2011 Dec 15;6(12):e28809. doi: 10.1371/journal.pone.0028809 (PMC3240631; doi:10.1371/journal.pone.0028809)

Fig. S1. Frequency distribution of the number of extrapair offspring, EPO (n= 130) across males (n= 59). The numbers in/above each bar refer to sample size. This distribution can be interpreted in two ways: (i) as a bimodal trait with nadir at two EPO (for which there are additional biological reasons, see Methods section in the main text), or (ii) as a negative binomial trait.

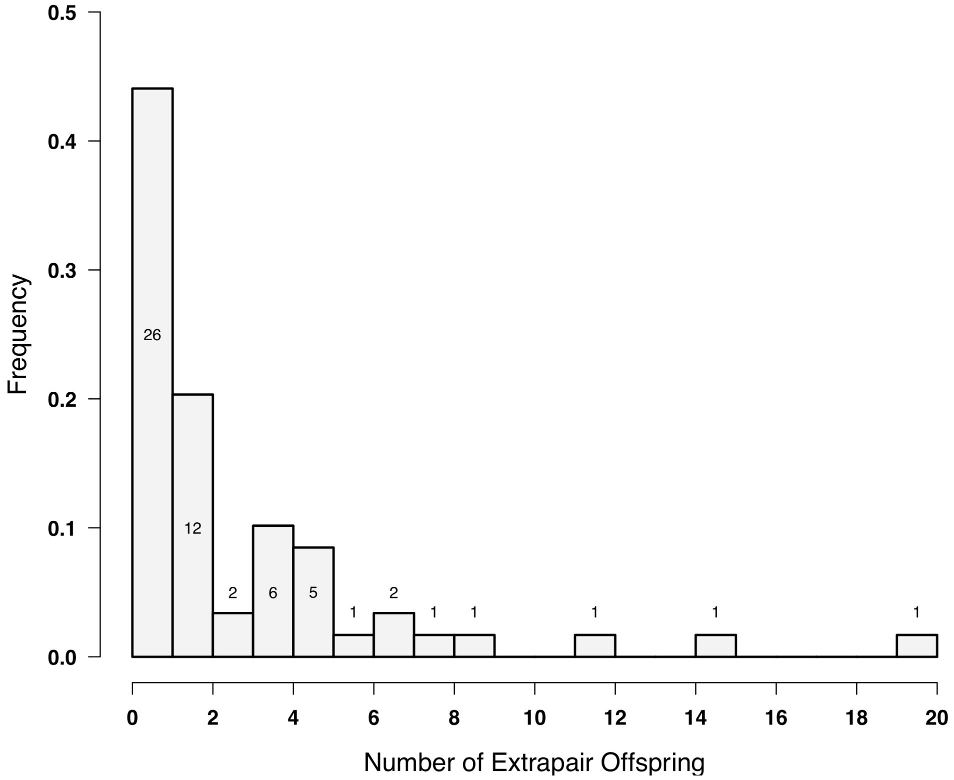

Supplement: Figure S1 — Frequency distribution of number of extrapair offspring per male. (PDF) [file pone.0028809.s001.pdf]
