# Supplementary material for: Maintenance of Sperm Variation in a Highly Promiscuous Wild Bird
Source: PLoS One. 2011 Dec 15;6(12):e28809. doi: 10.1371/journal.pone.0028809 (PMC3240631; doi:10.1371/journal.pone.0028809)

Fig. S2. Frequency distribution of the number of sperm measured per male (n= 59 males). The numbers in/above each bar refers to sample size. Forty-six males have at least five sperm measured.

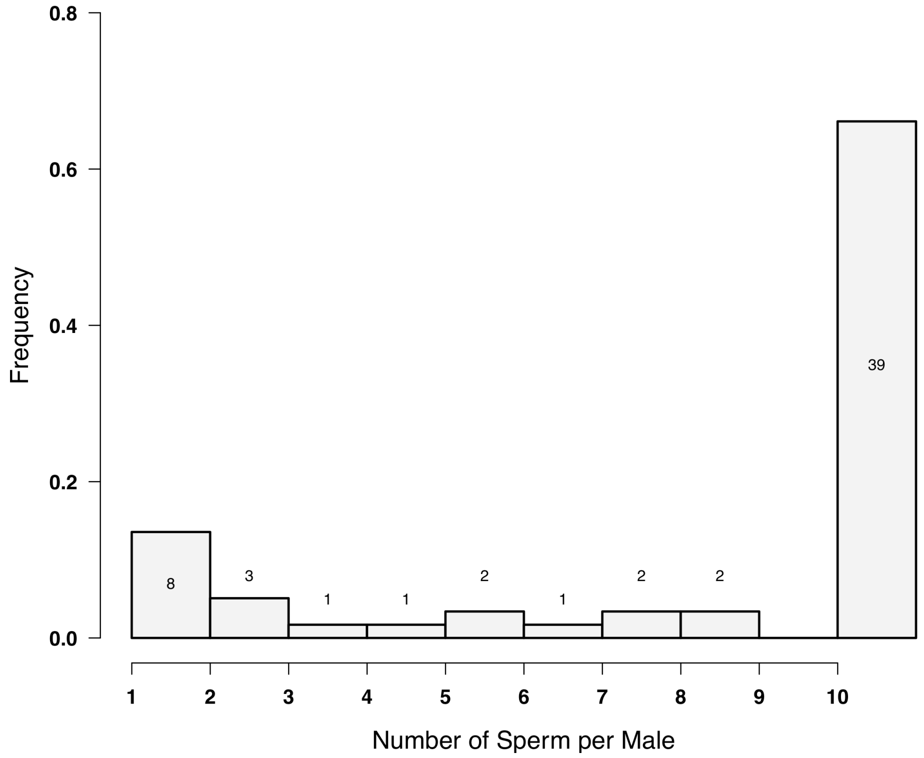

Supplement: Figure S2 — Frequency distribution of the number of sperm measured per male. (PDF) [file pone.0028809.s002.pdf]
