# Supplementary material for: Maintenance of Sperm Variation in a Highly Promiscuous Wild Bird
Source: PLoS One. 2011 Dec 15;6(12):e28809. doi: 10.1371/journal.pone.0028809 (PMC3240631; doi:10.1371/journal.pone.0028809)
Supplement: Figure S3 — Assessment of the accuracy of individual sperm morphometry estimates obtained using different numbers of sperm per male. (PDF) [file pone.0028809.s003.pdf]

**Figure S3 (next page).** Assessment of the accuracy of individual sperm morphometry estimates obtained using different numbers of sperm per male. A modified version of the method described in [1] was used. Briefly, we assessed individual mean trait estimate accuracy as the fit ($R^2$) of linear regressions of mean sperm morphometry obtained using n= 1 to n= 9 sperm per male (sampling without repetition) against each individual's 'accurate' estimate (i.e. that obtained with all 10 sperm; n= 39 males). The bootstrapped accuracy values (mean ± sd from 1000 repeats) for all sperm traits are plotted below. Note that across all sperm traits, c. 70 % of intramale variance can be captured by measuring a single sperm. Therefore, even these low sperm number samples can provide a biologically meaningful mean trait value.

**References**

**[1]** Pattarini JA, Starmer WT, Bjork A, Pitnick S (2006) Mechanisms underlying the sperm quality advantage in Drosophila melanogaster. Evolution 60: 2064–2080.

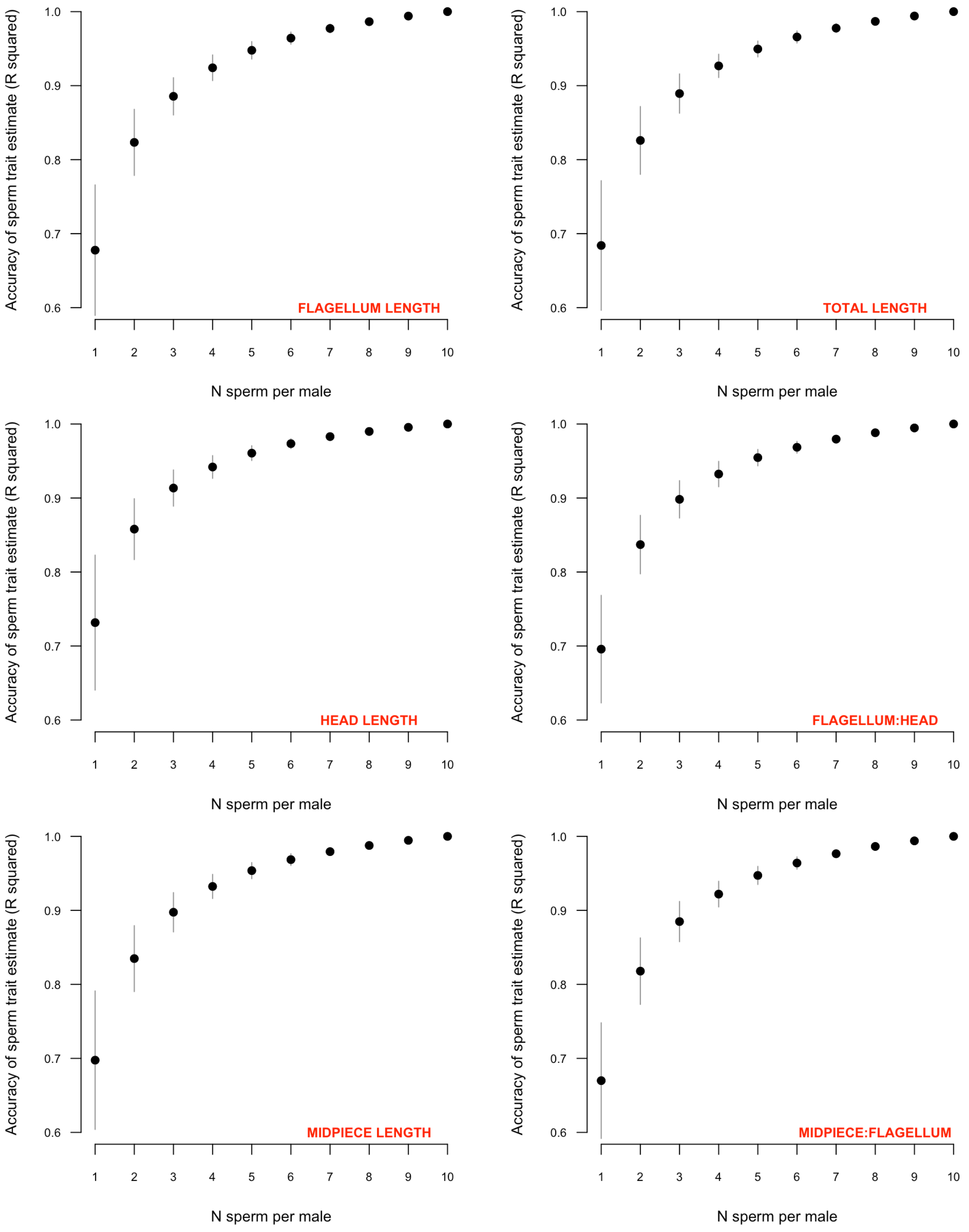
Accuracy of sperm trait estimate (R squared)
N sperm per male
FLAGELLUM LENGTH
TOTAL LENGTH
HEAD LENGTH
FLAGELLUM:HEAD
MIDPIECE LENGTH
MIDPIECE:FLAGELLUM
1.0
0.9
0.8
0.7
0.6
1
2
3
4
5
6
7
8
9
10
