# Supplementary material for: Maintenance of Sperm Variation in a Highly Promiscuous Wild Bird
Source: PLoS One. 2011 Dec 15;6(12):e28809. doi: 10.1371/journal.pone.0028809 (PMC3240631; doi:10.1371/journal.pone.0028809)
Supplement: Table S1 — Review of the evidence for paternity success trade-offs in male (pre-copulatory) phenotype in birds. (PDF) [file pone.0028809.s004.pdf]

Table S1. Review of the evidence for paternity success trade-offs in male (pre-copulatory) phenotype in birds. Only those studies that report *simultaneously* but also give *independent measures* of the effects of a given phenotype in a male's within- and extrapair paternity success are included. Incidence of extrapair paternity, EPP, is the percentage of broods with at least one extrapair young observed in the species (obtained from [2], when populations levels were not provided in the cited reference). Antagonistic selection occurs when the same phenotypic trait is under opposing directional selection with respect to extrapair and within-pair paternity success.

Species	Male phenotypic trait	Shape of Selection		Reference	EPP
		Extrapair success	Within-pair success		
Carpodacus erythrinus	Plumage	Positive	None	[3]	32%
Carpodacus mexicanus	Song	Positive	Positive	[4]	14%
Cyanistes caeruleus	Song, Age	Positive	Positive	[5]	40%
	Plumage (manipulated)	Positive	None	[6]	
	Plumage (natural)	Negative	Positive	[7]	
Emberiza schoeniclus	Exploratory behavior, Age	Positive	None	[8]	64%
Ficedula albicollis	Plumage	Positive	Positive	[9]	33%
Ficedula hypoleuca	Plumage (UV)	None	Positive	[10]	13%
Geothlypis trichas	Plumage, Age	Positive	None	[11]	49%
	Song, Condition	None	None		
Hirundo rustica	Tail length (natural & artificial)	Positive	Positive	[12]	n/a
Icterus galbula bullockii	Age	Positive	Positive	[13]	46%
	Condition	None	None		
Junco hyemalis	Testosterone levels (artificial)	Stabilizing	Positive	[14]	n/a
Luscinia svecica	Plumage	Positive or None	Positive	[15]	51%
	Size, Condition	None	None		
	Age	Positive	None		
Malurus cyaneus	Plumage molt date	Positive	None	[16]	95%
	Sperm morphology	Negative	Positive	This study	
Malurus melanocephalus	Plumage	Positive	None	[17]	51%
Pachycephala pectoralis	Song	Positive	Positive	[18]	23%
	Plumage	Positive	None		
Periparus ater	Age	Positive	Positive	[19]	67%
Phylloscopus fuscatus	Song	Positive	Positive	[20]	59%
Progne subis	Age	Positive	Positive	[21]	n/a
Promerops cafer	Tail length	Positive	Negative	[22]	71%
Setophaga ruticilla	Age	Positive	Positive	[23]	59%
Tyrannus tyrannus	Song, Size	Positive	Positive	[24]	61%
Wilsonia citrina	Age, Size	None	None	[25]	35%
	Song	Positive	Positive (indirect)	[26]	
	Plumage	None	None		

References

[2] Griffith SC, Owens IPF, Thuman KA (2002) Extra pair paternity in birds: a review of interspecific variation and adaptive function. Mol Ecol 11: 2195–2212.

[3] Albrecht T, Vinkler M, Schnitzer J, Polakova R, Munclinger P, et al. (2009) Extra-pair fertilizations contribute to selection on secondary male ornamentation in a socially monogamous passerine. J Evol Biol 22: 2020–2030.

[4] Mennill D, Badyaev A, Jonart L, Hill G (2006) Male house finches with elaborate songs have higher reproductive performance. Ethology 112: 174–180.

[5] Kempenaers B, Verheyren GR, Dhondt AA (1997) Extrapair paternity in the blue tit (Parus caeruleus): female choice, male characteristics, and offspring quality. Behav Ecol 8: 481–492.

[6] Delhey K, Peters A, Kempenaers B (2007) Cosmetic coloration in birds: Occurrence, function, and evolution. Am Nat 169: S145–S158.

[7] Delhey K, Johnsen A, Peters A, Andersson S, Kempenaers B (2003) Paternity analysis reveals opposing selection pressures on crown coloration in the blue tit (Parus caeruleus). Proc R Soc Lond B 270: 2057–2063.

[8] Kleven O, Marthinsen G, Lifjeld J (2006) Male extraterritorial forays, age and paternity in the socially monogamous reed bunting (Emberiza schoeniclus). J Ornithol 147: 468–473.

[9] Sheldon B, Ellegren H (1999) Sexual selection resulting from extrapair paternity in collared flycatchers. Anim Behav 57: 285–298.

[10] Lehtonen PK, Primmer CR, Laaksonen T (2009) Different traits affect gain of extrapair paternity and loss of paternity in the pied flycatcher, Ficedula hypoleuca. Anim Behav 77: 1103–1110.

[11] Thusius K, Peterson K, Dunn P, Whittingham L (2001) Male mask size is correlated with mating success in the common yellowthroat. Anim Behav 62: 435–446.

[12] Saino N, Primmer C, Ellegren H, Møller A (1997) An experimental study of paternity and tail ornamentation in the barn swallow (Hirundo rustica). Evolution 51: 562–570.

[13] Richardson D, Burke T (1999) Extra-pair paternity in relation to male age in Bullock's orioles. Mol Ecol 8: 2115–2126.

[14] Mcglothlin JW, Whittaker DJ, Schrock SE, Gerlach NM, Jawor JM, et al. (2010) Natural selection on testosterone production in a wild songbird population. Am Nat 175: 687–701.

[15] Johnsen A, Lifjeld JT, Andersson S, Ornborg J, Amundsen T (2001) Male characteristics and fertilisation success in bluethroats. Behaviour 138: 1371–1390.

[16] Dunn PO, Cockburn A (1999) Extrapair mate choice and honest signaling in cooperatively breeding superb fairy-wrens. Evolution 53: 938–946.

[17] Webster MS, Varian CW, Karubian J (2008) Plumage color and reproduction in the red-backed fairy-wren: Why be a dull breeder? Behav Ecol 19: 517–524.

[18] Dongen W, Mulder R (2009) Multiple ornamentation, female breeding synchrony, and extra-pair mating success of golden whistlers (Pachycephala pectoralis). J Ornithol 150: 607–620.

[19] Schmoll T, Mund V, Dietrich-Bischoff V, Winkel W, Lubjuhn T (2007) Male age predicts extrapair and total fertilization success in the socially monogamous coal tit. Behav Ecol 18: 1073–1081.

[20] Forstmeier W (2002) Factors contributing to male mating success in the polygynous dusky warbler (Phylloscopus fuscatus). Behaviour 139: 1361–1381.

[21] Wagner R, Schug M, Morton E (1996) Condition dependent control of paternity by female purple martins: Implications for coloniality. Behav Ecol Sociobiol 38: 379–389.

[22] Mcfarlane ML, Evans MR, Feldheim KA, Preault M, Bowie RC, et al. (2010) Long tails matter in sugarbirds--positively for extrapair but negatively for within-pair fertilization success. Behav Ecol 21: 26–32.

[23] Perreault S, Lemon R, Kuhnlein U (1997) Patterns and correlates of extrapair paternity in American redstarts (Setophaga ruticilla). Behav Ecol 8: 612–621.

[24] Dolan AC, Murphy MT, Redmond LJ, Sexton K, Duffield D (2007) Extrapair paternity and the opportunity for sexual selection in a socially monogamous passerine. Behav Ecol 18: 985–993.

[25] Stutchbury B, PIPER W, Neudorf D, Tarof S, Rhymer J, et al. (1997) Correlates of extra-pair fertilization success in hooded warblers. Behav Ecol Sociobiol 40: 119–126.

[26] Chiver I, Stutchbury B, Morton E (2008) Do male plumage and song characteristics influence female off-territory forays and paternity in the hooded warbler? Behav Ecol Sociobiol 62: 1981–1990.
