# Supplementary material for: Maintenance of Sperm Variation in a Highly Promiscuous Wild Bird
Source: PLoS One. 2011 Dec 15;6(12):e28809. doi: 10.1371/journal.pone.0028809 (PMC3240631; doi:10.1371/journal.pone.0028809)
Supplement: Table S2 — Repeatability of sperm measurements. (PDF) [file pone.0028809.s005.pdf]

Table S2. Repeatability of the measurements and of the mean based on the LMM.REML method for Gaussian data ([27]; R package rptR). Coefficients of variation, CV, for sperm traits were calculated as follows: (i) intermale CV using n=46 males with at least 5 sperm each ([28]), and (ii) intramale CV using n=10 males with 50 sperm each ([29]).

	**Repeatability of the measurement**			**Repeatability of the mean**	**Intermale CV**	**Intramale CV**
	**R**	**C.I.**	**p**	**r**		
**Head Length**	0.68	0.57-0.75	<0.0001	0.94	4.1	2.3
**Flagellum Length**	0.65	0.53-0.73	<0.001	0.93	2.3	1.4
**(Straight) Midpiece Length**	0.54	0.41-0.64	<0.0001	0.90	3.5	3.3
**Total Length**	0.68	0.57-0.76	<0.0001	0.94	2.1	1.1
**Flagellum:Head ratio**	0.61	0.48-0.70	<0.0001	0.92	4.5	3.0
**Midpiece:Flagellum ratio**	0.55	0.43-0.65	<0.0001	0.90	3.7	3.2

References

[27] Nakagawa S, Schielzeth H (2010) Repeatability for Gaussian and non-Gaussian data: a practical guide for biologists. Biol Rev 85: 935–956.
[28] Calhim S, Immler S, Birkhead TR, Pizzari T (2007) Postcopulatory Sexual Selection Is Associated with Reduced Variation in Sperm Morphology. PLoS ONE 2: e413.
[29] Immler S, Calhim S, Birkhead TR (2008) Increased postcopulatory sexual selection reduces the intramale variation in sperm design. Evolution 62: 1538–1543.
