# Supplementary material for: Maintenance of Sperm Variation in a Highly Promiscuous Wild Bird
Source: PLoS One. 2011 Dec 15;6(12):e28809. doi: 10.1371/journal.pone.0028809 (PMC3240631; doi:10.1371/journal.pone.0028809)
Supplement: Table S3 — Effects of status, age and group type in sperm morphometrics. (PDF) [file pone.0028809.s006.pdf]

Table S3. No evidence for the effect of status (dominant vs. subordinate), age, or group type (cooperative breeder vs. monogamous pair) in sperm morphometrics. (MANOVA test).

	**Pillai**	**Approx F**	**P**	**d.f.**
**Status**	0.114	1.047	0.40	1, 54
**Age**	0.144	1.379	0.24	1, 54
**Group Type**	0.198	0.913	0.54	2, 54
