# Supplementary material for: Maintenance of Sperm Variation in a Highly Promiscuous Wild Bird
Source: PLoS One. 2011 Dec 15;6(12):e28809. doi: 10.1371/journal.pone.0028809 (PMC3240631; doi:10.1371/journal.pone.0028809)
Supplement: Table S4 — Full statistical outputs. (PDF) [file pone.0028809.s007.pdf]

Table S4. Full Statistical Outputs

i) Cuckolding Success (categorical variable) ~ Composite Sperm Traits

[full dataset]

Dependent Variable: High success (≥2 EPO sired) vs. Low Success (≤1 EPO sired)
Model Type: GLM with quasibinomial errors, weighed by number of sperm measured
Sample N: 59 males

	estimate	s.e.	z	p
**(Intercept)**	169.36	83.39	2.03	0.047
**Seasons No.**	1.02	0.29	3.50	<0.001
**log(Total Length)**	-30.10	19.49	-1.54	0.128
**log(Flagellum:Head)**	-18.95	9.25	-2.05	0.045
**log(Midpiece:Flagellum)**	9.56	11.35	0.84	0.403

	estimate	s.e.	z	p
**(Intercept)**	23.42	13.59	1.72	0.090
**Seasons No.**	0.90	0.26	3.43	0.001
**log(Flagellum:Head)**	-18.60	9.23	-2.01	0.049

[restricted dataset to males with at least 5 sperm measured]

Dependent Variable: High success (≥2 EPO sired) vs. Low Success (≤1 EPO sired)
Model Type: GLM with quasibinomial errors
Sample N: 46 males

	estimate	s.e.	z	p
**(Intercept)**	188.34	99.11	1.90	0.0574
**Seasons No.**	0.99	0.33	3.05	0.003
**log(Total Length)**	-32.14	22.11	-1.45	0.146
**log(Flagellum:Head)**	-21.65	11.32	-1.91	0.056
**log(Midpiece:Flagellum)**	13.57	14.07	0.97	0.335

	estimate	s.e.	z	p
**(Intercept)**	24.87	15.29	1.63	0.104
**Seasons No.**	0.88	0.27	3.19	0.001
**log(Flagellum:Head)**	-19.50	10.40	-1.88	0.061

[restricted dataset to males with complete lifetime paternity data]

Dependent Variable: High success (≥2 EPO sired) vs. Low Success (≤1 EPO sired)
Model Type: GLM with quasibinomial errors, weighed by number of sperm measured
Sample N: 47 males

	estimate	s.e.	z	p
**(Intercept)**	25.74	14.35	1.79	0.080
**Seasons No.**	0.93	0.26	3.60	0.001
**log(Flagellum:Head)**	-19.80	9.74	-2.03	0.048

ii) Cuckolding Success (categorical variable) ~ Independent Sperm Traits

[full dataset]

Dependent Variable: High success (≥2 EPO sired) vs. Low Success (≤1 EPO sired)
Model Type: GLM with quasibinomial errors, weighed by number of sperm measured
Sample N: 59 males

	estimate	s.e.	z	p
**(Intercept)**	156.22	74.58	2.09	0.041
**Seasons No.**	1.02	0.29	3.51	<0.001
**log(Head Length)**	13.36	9.89	1.35	0.182
**log(Flagellum Length)**	-53.35	20.07	-2.66	0.010
**log(SHL)**	9.58	11.37	0.84	0.403

	estimate	s.e.	z	p
**(Intercept)**	182.89	68.87	2.66	0.010
**Seasons No.**	0.98	0.27	3.64	<0.001
**log(Flagellum Length)**	-44.83	16.57	-2.71	0.009

[restricted dataset to males with at least 5 sperm measured]

Dependent Variable: High success (≥2 EPO sired) vs. Low Success (≤1 EPO sired)
Model Type: GLM with quasibinomial errors
Sample N: 46 males

	estimate	s.e.	z	p
**(Intercept)**	174.33	89.32	1.95	0.051
**Seasons No.**	0.99	0.33	3.05	0.003
**log(Head Length)**	15.70	11.78	1.33	0.182
**log(Flagellum Length)**	-61.80	26.59	-2.32	0.0201
**log(SHL)**	13.65	14.11	0.97	0.333

	estimate	s.e.	z	p
**(Intercept)**	189.66	84.70	2.24	0.025
**Seasons**	0.92	0.30	3.05	0.003
**log(Flagellum Length)**	-46.38	20.34	-2.28	0.023

[restricted dataset to males with complete lifetime paternity data]

Dependent Variable: High success (≥2 EPO sired) vs. Low Success (≤1 EPO sired)
Model Type: GLM with quasibinomial errors, weighed by number of sperm measured
Sample N: 47 males

	estimate	s.e.	z	p
**(Intercept)**	149.56	62.43	2.40	0.021
**Seasons No.**	0.94	0.25	3.74	<0.001
**log(Flagellum Length)**	-36.70	14.99	-2.45	0.018

iii) An alternative: Cuckolding Success (numerical variable) ~ Composite Sperm Traits

[full dataset]

Dependent Variable: Number of extrapair offspring
Model Type: GLM with negative binomial errors, weighed by number of sperm measured
Sample N: 59 males

	estimate	s.e.	z	p
**(Intercept)**	41.34	24.98	1.66	0.098
**Seasons No.**	0.41	0.05	7.50	<0.001
**log(Total Length)**	-6.25	5.96	-1.05	0.294
**log(Flagellum:Head)**	-5.20	2.52	-2.07	0.039
**log(Midpiece:Flagellum)**	5.12	3.30	1.55	0.120

	estimate	s.e.	z	p
**(Intercept)**	6.47	3.74	1.73	0.083
**Seasons No.**	0.40	0.06	7.31	<0.001
**log(Flagellum:Head)**	-5.16	2.48	-2.08	0.038

iv) An alternative: Cuckolding Success (numerical variable) ~ Independent Sperm Traits

[full dataset]

Dependent Variable: Number of extrapair offspring
Model Type: GLM with negative binomial errors, weighed by number of sperm measured
Sample N: 59 males

	estimate	s.e.	z	p
**(Intercept)**	38.65	22.26	1.74	0.083
**Seasons No.**	0.41	0.05	7.50	<0.001
**log(Head Length)**	4.03	2.82	1.43	0.153
**log(Flagellum Length)**	-15.49	5.35	-2.90	0.004
**log(SHL)**	5.13	3.29	1.56	0.119

	estimate	s.e.	z	p
**(Intercept)**	45.60	20.90	2.18	0.029
**Seasons**	0.40	0.06	7.21	<0.001
**log(Flagellum Length)**	-11.18	4.99	-2.24	0.025

v) Cuckolding Defense ~ Composite Sperm Traits

[full dataset]

Dependent Variable: Proportion of own nestlings sired
Model Type: GLMM with binomial errors, weighed by number of sperm measured, with male identity as random factor
Sample N: 255 broods of 47 males

	estimate	s.e.	z	p
**(Intercept)**	-68.94	39.93	-1.73	0.084
**Helper No.**	0.12	0.10	1.23	0.217
**log(Total Length)**	10.49	9.55	1.10	0.272
**log(Flagellum:Head)**	11.24	4.26	2.64	0.008
**log(Midpiece:Flagellum)**	-2.85	4.45	-0.64	0.523

	estimate	s.e.	z	p
**(Intercept)**	-19.10	6.52	-2.93	0.003
**log(Flagellum:Head)**	11.50	4.31	2.67	0.008

[restricted dataset to males with complete lifetime paternity data]

Dependent Variable: Proportion of own nestlings sired
Model Type: GLMM with binomial errors, weighed by number of sperm measured, with male identity as random factor
Sample N: 141 broods of 30 males

	estimate	s.e.	z	p
**(Intercept)**	-31.11	11.03	-2.82	0.005
**log(Flagellum:Head)**	19.18	7.23	2.65	0.008

[restricted dataset to males with at least 5 sperm measured]

Dependent Variable: Proportion of own nestlings sired
Model Type: GLMM with binomial errors, with male identity as random factor
Sample N: 208 broods of 36 males

	estimate	s.e.	z	p
**(Intercept)**	-50.12	42.81	-1.17	0.242
**Helper No.**	0.13	0.13	0.95	0.34
**log(Total Length)**	7.91	10.10	0.79	0.43
**log(Flagellum:Head)**	7.29	4.19	1.74	0.081
**log(Midpiece:Flagellum)**	-1.85	4.79	-0.39	0.700

	estimate	s.e.	z	p
**(Intercept)**	-12.58	6.47	-1.94	0.052
**log(Flagellum:Head)**	7.26	4.27	1.70	0.089

vi) Cuckolding Defense ~ Independent Sperm Traits

[full dataset]

Dependent Variable: Proportion of own nestlings sired

Model Type: GLMM with binomial errors, weighed by number of sperm measured, with male identity as random factor

Sample N: 255 broods of 47 males

	estimate	s.e.	z	p
**(Intercept)**	-63.97	35.53	-1.80	0.072
**Helper No.**	0.12	0.10	1.24	0.217
**log(Head Length)**	-9.31	4.82	-1.93	0.053
**log(Flagellum Length)**	22.66	8.39	2.70	0.007
**log(SHL)**	-2.86	4.45	-0.64	0.520

	estimate	s.e.	z	p
**(Intercept)**	-85.90	34.87	-2.46	0.014
**log(Flagellum Length)**	20.08	8.32	2.45	0.016

[restricted dataset to males with complete lifetime paternity data]

Dependent Variable: Proportion of own nestlings sired

Model Type: GLMM with binomial errors, weighed by number of sperm measured, with male identity as random factor

Sample N: 141 broods of 30 males

	estimate	s.e.	z	p
**(Intercept)**	-94.96	42.77	-2.22	0.026
**log(Flagellum Length)**	22.18	10.19	2.18	0.030

[restricted dataset to males with at least 5 sperm measured]

Dependent Variable: Proportion of own nestlings sired

Model Type: GLMM with binomial errors, with male identity as random factor

Sample N: 208 broods of 36 males

	estimate	s.e.	z	p
**(Intercept)**	-46.33	38.16	-1.21	0.225
**Helper No.**	0.13	.013	0.95	0.342
**log(Head Length)**	-5.83	4.68	-1.25	0.21
**log(Flagellum Length)**	15.60	8.96	1.74	0.082
**log(SHL)**	-1.87	4.78	-0.39	0.696

	estimate	s.e.	z	p
**(Intercept)**	-59.90	36.95	-1.62	0.105
**log(Flagellum Length)**	13.90	8.81	1.58	0.114
