# Supplementary material for: Maintenance of Sperm Variation in a Highly Promiscuous Wild Bird
Source: PLoS One. 2011 Dec 15;6(12):e28809. doi: 10.1371/journal.pone.0028809 (PMC3240631; doi:10.1371/journal.pone.0028809)
Supplement: Table S5 — Effect sizes. (PDF) [file pone.0028809.s008.pdf]

Table S5. Effect Sizes

We obtained effect sizes and their 95% confidence intervals, CIs, by repeating the minimal adequate models using standardized variables (see [30,31] for more details). For these analyses in particular, we used the probit link function for the (quasi)binomial error structure and subtracted the mean from the predictor variable values (z-score transformation). The model term estimate (slopes) from these "single linear regression" or "multiple linear regression" models can be used as effect sizes. Note that the required no correlation between covariates in multiple-predictor models is observed (season number is not correlated with sperm morphology; see Table S3; flagellum length rho= -0.04, p> 0.7; flagellum:head ratio: rho= 0.05, p> 0.7). Therefore, one can use of the sperm trait slope as standardized effect size despite multiple predictors (see [30]). Confidence intervals were obtained from parameter standard error multiplied either by (i) a value calculated based on t distribution and model degrees of freedom with the R function qt(0.975, df) for GLMs or (ii) the constant 1.96 for GLMMs (equivalent z distribution).

References

[30] Schielzeth H (2010) Simple means to improve the interpretability of regression coefficients. Methods Ecol Evol 1: 103–113.

[31] Nakagawa S, Cuthill IC (2007) Effect size, confidence interval and statistical significance: a practical guide for biologists. Biol Rev 82: 591–605.

Response	Predictor	Model Type	Effect size	95% CI
Cuckolding Success	Flagellum Length	Weighed	-0.40	-0.67, -0.12
(controlling for season number)		≥ 5 sperm	-0.41	-0.73, -0.10
		Lifetime, weighed	-0.33	-0.64, -0.03
Cuckolding Success	Flagellum:Head Ratio	Weighed	-2.38	-4.65, -0.12
(controlling for season number)		≥ 5 sperm	-2.47	-5.15, 0.20
		Lifetime, weighed	-2.64	-5.56, 0.28
Cuckolding Defense	Flagellum Length	Weighed	0.17	0.04, 0.31
(male identity included as a random factor)		≥ 5 sperm	0.12	-0.03, 0.26
		Lifetime, weighed	0.19	0.02, 0.36
Cuckolding Defense	Flagellum:Head Ratio	Weighed	1.47	0.42, 2.53
(male identity included as a random factor)		≥ 5 sperm	0.91	-0.13, 1.95
		Lifetime, weighed	2.33	0.67, 3.99
